# Association between body shape index and risk of mortality in the United States

**DOI:** 10.1038/s41598-022-15015-x

**Published:** 2022-07-04

**Authors:** Heysoo Lee, Hye Soo Chung, Yoon Jung Kim, Min Kyu Choi, Yong Kyun Roh, Wankyo Chung, Jae Myung Yu, Chang-Myung Oh, Shinje Moon

**Affiliations:** 1 Department of Family Medicine, Hallym University College of Medicine, Chuncheon, Korea; 2 Division of Endocrinology and Metabolism, Department of Internal Medicine, Hallym University College of Medicine, Chuncheon, 07441 Seoul Korea; 3 Department of Public Health Science, Graduate School of Public Health, Seoul National University, Seoul, Korea; 4 Department of Biomedical Science and Engineering, Gwangju Institute of Science and Technology, Gwangju, 61005 Korea

**Keywords:** Cardiology, Endocrinology

## Abstract

The body mass index (BMI) neither differentiates fat from lean mass nor does it consider adipose tissue distribution. In contrast, the recently introduced z-score of the log-transformed A Body Shape Index (LBSIZ) can be applied to measure obesity using waist circumference (WC), height, and weight. We aimed to investigate the association between LBSIZ and mortality. We used data from the National Health and Nutrition Examination Survey 1999–2014 and linked the primary dataset to death certificate data from the National Death Index with mortality follow-up through December 31, 2015. A multiple Cox regression analysis was performed to evaluate the hazard ratio (HR) of all-cause and cardiovascular disease (CVD) mortalities with adjustment for baseline characteristics. LBSIZ, WC, and BMI showed positive association with total fat percentage (P < 0.001); however, only WC and BMI were positively associated with appendicular skeletal mass index (ASMI) (P < 0.001). In the multiple Cox regression analysis, only LBSIZ showed a significant HR for all-cause and CVD mortalities. Under restricted cubic spline regression, mortality risk increased with LBSIZ. However, BMI and WC showed a U-shape association. In conclusion, LBSIZ is strongly associated with all-cause and CVD mortalities. Since LBSIZ is independent of BMI, LBSIZ complements BMI to identify high-risk groups for mortality even in individuals with low or normal BMI.

## Introduction

According to the World Health Organization, 13% of the world’s population in 2016 were obese, and the prevalence of obesity has increased dramatically by approximately threefold in the last 30 years^1^. Obesity is a risk factor for several diseases, including cardiovascular disease (CVD), diabetes, cerebral infarction, and cancer, and is associated with approximately 4.8% of mortality worldwide^2–6^. Such increasing prevalence and clinical significance suggest the importance of accurate diagnosis of obesity.

Non-invasive tests performed for the accurate measurement of body fat include computed tomography (CT), magnetic resonance imaging, dual-energy X-ray absorptiometry (DEXA), and positron emission tomography/CT; however, these tests are associated with a high-cost burden and low accessibility. Therefore, body mass index (BMI), which is calculated using a simple formula of weight divided by square of the height (kg/m^2^), has been used to define obesity. However, BMI does not differentiate between muscle and fat, and thus is inaccurate in estimating body fat content^7^. As a result, it is not unexpected that BMI is limited in predicting the risk of CVD, diabetes, cerebral infarction, cancer, and death^8–10^.

A Body Shape Index (ABSI), which is a power law expressing waist circumference (WC)/expected value for WC, considering weight and height (i.e. BMI), was derived from the US National Health and Nutrition Examination Survey (NHANES). ABSI adjusts WC for BMI so that ABSI and BMI will become statistically independent^11^. In a 2018 meta-analysis study, ABSI was associated with high blood pressure, diabetes, CVD, and its association with mortality was stronger than that of BMI or WC^12^. As initially described, ABSI is sex dependent (higher in males than females) and increases with age. It was suggested that z scores be computed with adjustments for gender and age. An important finding has shown that, unlike BMI, ABSI has been applicable across a range of populations and demographics. As with BMI, scaling exponent adjustments customized to other populations demonstrated only small differences^13^. However, the skewness and lack of cut-off values has been said to limit the identification of the high-risk groups for obesity-related diseases, and the scaling exponents may differ by ethnicity^14,15^. The use of ABSI in clinical settings has been suggested^16,17^, but remains largely preclinical and has not been documented in the literature. Alternatively, the use of Z scores, log-transformed A Body Shape Index (LBSIZ) has been suggested to complement ABSI with respect to the noted limitations^14,18^. LBSIZ can predict hypertension and health-related quality of life^15^ and is associated with the risk of CVDs in the Korean population. Furthermore, in a cross-sectional study using the NHANES, LBSIZ was significantly correlated with the risk of CVDs^19^. However, there are no longitudinal studies on LBSIZ outside Korea.

Therefore, this study aimed to investigate the association between LBSIZ and risk of all-cause and CVD mortalities using the NHANES, a representative sample of the United States population, from which ABSI was initially derived.

## Results

### Baseline characteristics

**Figure 1:**
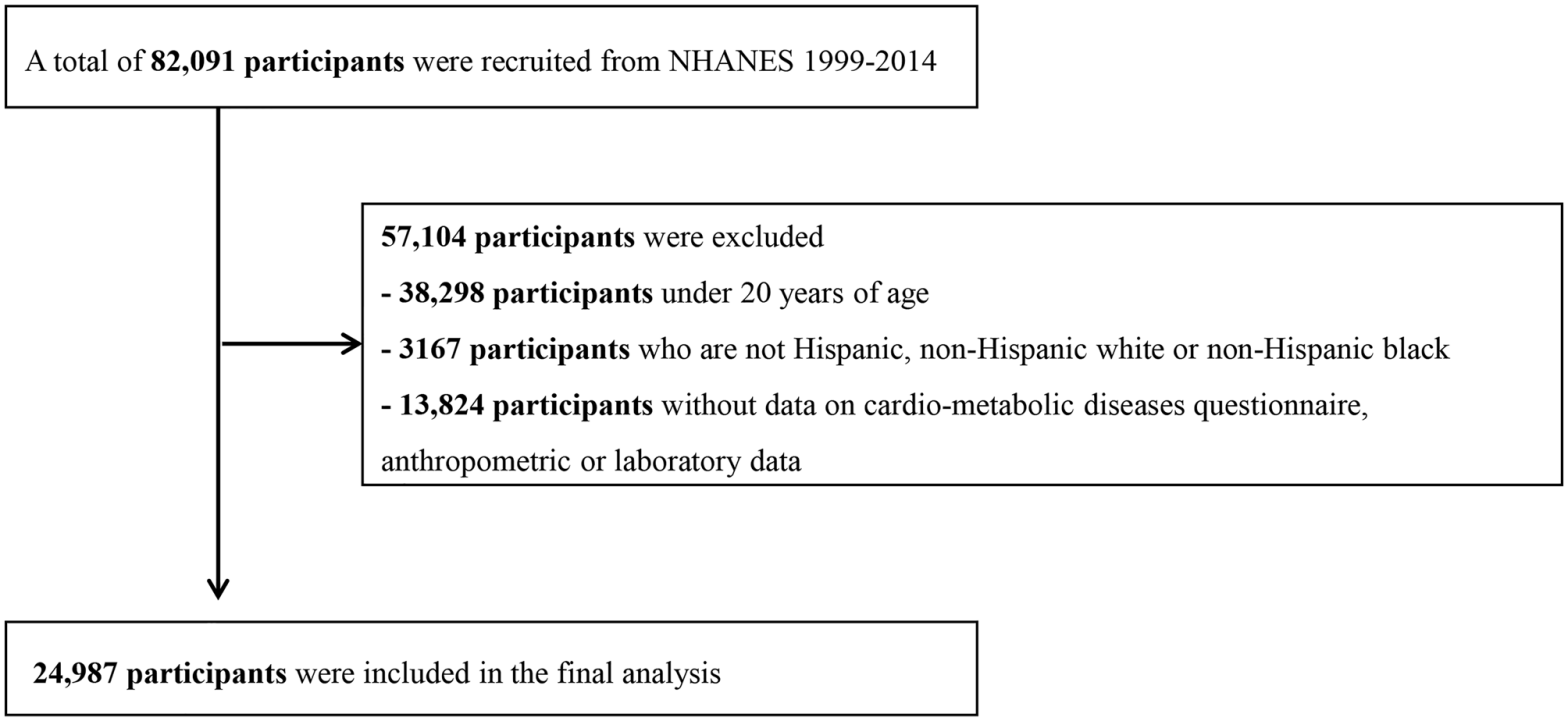
Flowchart for final selection.

In total, 24,987 adults aged 20–85 years were included in this study (Fig. 1). The mean age of the participants was 52.9 ± 17.4 years, and 51.7% of them were women. The mean BMI and WC values, respectively, were 28.9 ± 5.7 kg/m^2^ (range 15.4–74.1) and 102.9 ± 14.9 cm (range 61.8–178.2) in men, and 29.7 ± 5.2 kg/m^2^ (range 13.4–82.1) and 98.1 ± 15.9 cm (range 58.8–172.5) in women. In total, 3,355 participants died during a mean follow-up period of 90.6 ± 53.5 months (median: 83 months). Of those, 755 deaths were due to CVD. Table 1 shows the demographic, anthropometric, and clinical characteristics according to the all-cause mortality of the participants. The mean age, proportion of males, smokers, and drinkers were higher in those who died than in survivors. In addition, systolic blood pressure, fasting glucose, hemoglobin A1c (HbA1c), and triglyceride levels were higher, whereas high-density lipoprotein-cholesterol level was lower in participants who died. The prevalence rates of previous CVD, diabetes mellitus, hypertension, and dyslipidemia were also higher in this group. Appendicular skeletal mass index (ASMI) was significantly lower in participants who died due to CVD; however, there was no significant difference in total fat percentage. The mean BMI of participants who died was 28.4 ± 6.1 kg/m^2^ (range 13.4–64.7), and it was significantly lower in the survivors (p < 0.001) at 29.5 ± 6.6 kg/m^2^ (range 14.2–82.1). However, the obesity parameters WC and LBSIZ were higher in those who died than in survivors.

**Table 1:** Characteristics of subjects according to all-cause mortality.

	Alive	Death	p
	(N = 21,632)	(N = 3355)	
Age, years	50.3 ± 16.5	70.0 ± 13.1	< 0.001
Male sex, n (%)	10,179 (47.1%)	1888 (56.3%)	< 0.001
**Race/ethnicity, n (%) **			< 0.001
Mexican American	3590 (16.6%)	507 (15.1%)	
Other Hispanic	1860 ( 8.6%)	130 ( 3.9%)	
Non-Hispanic Black	11,524 (53.3%)	2145 (63.9%)	
Non-Hispanic White	4658 (21.5%)	573 (17.1%)	
Smokers, n (%)	9931 (45.9%)	2025 (60.4%)	< 0.001
Drinkers, n (%)	10,366 (47.9%)	1100 (32.8%)	< 0.001
History of cancer, n (%)	1968 (9.1%)	772 (23.1%)	< 0.001
BMI, kg/m^2^	29.5 ± 6.6	28.4 ± 6.1	< 0.001
WC, cm	100.2 ± 15.7	101.6 ± 15.2	< 0.001
LBSIZ	− 0.0 ± 1.0	0.7 ± 0.9	< 0.001
Systolic BP, mmHg	124.0 ± 18.0	136.7 ± 23.0	< 0.001
Diastolic BP, mmHg	70.8 ± 12.5	67.2 ± 15.9	< 0.001
Fasting glucose, mg/dL	107.3 ± 34.4	121.4 ± 51.0	< 0.001
HBA1c, %	5.7 ± 1.1	6.1 ± 1.3	< 0.001
Total cholesterol, mg/dL	201.8 ± 44.1	201.9 ± 48.3	0.945
Triglycerides, mg/dL	148.9 ± 139.7	161.4 ± 123.8	< 0.001
HDL-C, mg/dL	53.2 ± 16.1	52.4 ± 17.0	0.013
Previous CVD, n (%)	2111 (9.8%)	1206 (35.9%)	< 0.001
Diabetes Mellitus, n (%)	3747 (17.3%)	1105 (32.9%)	< 0.001
Hypertension, n (%)	10,500 (48.5%)	2758 (82.2%)	< 0.001
Dyslipidemia, n (%)	11,386 (52.6%)	2166 (64.6%)	< 0.001
CVD death		744 (22.2%)	
ASMI*	7.6 ± 1.5	7.1 ± 1.3	< 0.001
Total fat percentage*	34.3 ± 8.3	34.8 ± 7.8	0.059

*BMI* body mass index, *WC* waist circumference, *LBSIZ* z-score of the log-transformed A Body Shape Index, *HBA1c* hemoglobin A1c, *HDL-C* high-density lipoprotein cholesterol, *CVD* cardiovascular disease, *ASMI* appendicular skeletal mass index.

*Data was available from 1999 to 2006.

### Association between obesity parameters and body composition

BMI and WC were positively correlated with ASMI and total fat percentage. In contrast, LBSIZ was positively correlated with total fat percentage and negatively correlated with ASMI. Similar findings were observed following subgroup analysis by sex (Table 2).

**Table 2:** Correlation between obesity parameters with body composition (N-11780).

	ASMI	Total fat percentage
	Pearson correlation coefficient (p-value)	Pearson correlation coefficient (p-value)
**Total**		
BMI	0.585 (< 0.001)	0.557 (< 0.001)
WC	0.550 (< 0.001)	0.493 (< 0.001)
LBSIZ	− 0.117 (< 0.001)	0.082 (< 0.001)
**Men**		
BMI	0.799 (< 0.001)	0.651 (< 0.001)
WC	0.666 (< 0.001)	0.661 (< 0.001)
LBSIZ	− 0.361 (< 0.001)	0.501 (< 0.001)
**Women**		
BMI	0.819 (< 0.001)	0.818 (< 0.001)
WC	0.681 (< 0.001)	0.811 (< 0.001)
LBSIZ	-0.185 (< 0.001)	0.096 (< 0.001)

*BMI* body mass index, *WC* waist circumference, *LBSIZ* z-score of the log-transformed A Body Shape Index, *ASMI* appendicular skeletal mass index.

### Association between obesity parameters and mortality

In the multiple Cox regression analysis, an increase in LBSIZ was significantly associated with high all-cause mortality rate (hazard ratio [HR] 1.218, 95% confidence interval [CI] 1.169–1.270, P < 0.001), whereas BMI and WC had a negative association with all-cause mortality (BMI, HR: 0.982, 95% CI 0.976–0.989, P < 0.001; WC: 0.997, 95% CI 0.994–0.999, P = 0.018; Table 3). An increase in LBSIZ led to a significant increase in the risk of CVD death; however, BMI and WC were not associated with CVD mortality. In the subgroup analyses according to sex, an increase in LBSIZ was significantly associated with high all-cause and CVD mortalities regardless of sex (Table 3). In the analysis with propensity score matching (PSM) data^20^, an increase in LBSIZ led to a significant increase in the risk of all-cause and CVD death, whereas BMI had a negative association with all-cause and CVD mortalities (Table 3).

**Table 3:** Hazard ratio (95% confidence interval) for all-cause mortality and CVD mortality by each obesity parameter.

	All-cause mortality		CVD mortality	
	HR (95% CI)	P-value	HR (95% CI)	P-value
**Total**				
BMI	0.982 (0.976–0.989)	< 0.001	0.989 (0.975–1.004)	0.143
WC	0.997 (0.994–0.999)	0.018	1.000 (0.994–1.006)	0.984
LBSIZ	1.218 (1.169–1.270)	< 0.001	1.244 (1.138–1.360)	< 0.001
**Men**				
BMI	0.985 (0.975–0.995)	0.004	1.006 (0.987–1.026)	0.548
WC	0.998 (0.994–1.002)	0.310	1.005 (0.998–1.012)	0.191
LBSIZ	1.290 (1.206–1.379)	< 0.001	1.302 (1.138–1.491)	< 0.001
**Women**				
BMI	0.980 (0.971–0.989)	< 0.001	0.970 (0.948–0.991)	0.006
WC	0.996 (0.992–0.999)	0.025	0.992 (0.984–1.002)	0.103
LBSIZ	1.174 (1.114–1.237)	< 0.001	1.196 (1.062–1.347)	0.003
**PSM data**				
BMI	0.979 (0.972–0.986)	< 0.001	0.981 (0.966–0.997)	0.017
WC	0.996 (0.993–0.999)	0.006	0.998 (0.992–1.004)	0.519
LBSIZ	1.211 (1.160–1.264)	< 0.001	1.248 (1.139–1.368)	< 0.001

Adjusted for age, sex, ethnicity/race, smoking status, alcohol consumption, history of cancer at baseline, diabetes mellitus, hypertension, dyslipidemia, and previous CVD event.

*BMI* body mass index, *WC* waist circumference, *LBSIZ* z-score of the log-transformed A Body Shape Index, *CVD* cardiovascular disease.

In the subgroup analyses according to previous CVD status and history of cancer at the baseline survey, an increase in LBSIZ was significantly associated with high all-cause mortality, whereas BMI had a negative association regardless of underlying diseases (Table 4). An increase in LBSIZ led to a significant increase in the risk of CVD death; however, BMI and WC were not associated with CVD mortality in all subgroups with respect to previous CVD status and history of cancer (Table 4).

**Table 4:** Subgroup analysis of the association between each obesity parameter and mortality according to underlying diseases.

	All-cause mortality		CVD mortality	
	HR (95% CI)	P-value	HR (95% CI)	P-value
**Without previous CVD events**				
BMI	0.981 (0.973–0.989)	< 0.001	0.993 (0.974–1.012)	0.471
WC	0.996 (0.993–1.000)	0.026	1.003 (0.995–1.010)	0.526
LBSIZ	1.225 (1.165–1.289)	< 0.001	1.318 (1.169–1.486)	< 0.001
**With previous CVD**				
BMI	0.983 (0.972–0.994)	0.004	0.983 (0.963–1.005)	0.122
WC	0.997 (0.993–1.001)	0.190	0.996 (0.987–1.004)	0.319
LBSIZ	1.200 (1.118–1.287)	< 0.001	1.142 (1.001–1.303)	0.048
**Without history of cancer at baseline**				
BMI	0.985 (0.978–0.993)	< 0.001	0.993 (0.977–1.009)	0.378
WC	0.998 (0.995–1.001)	0.237	1.001 (0.995–1.007)	0.784
LBSIZ	1.223 (1.168–1.281)	< 0.001	1.221 (1.106–1.347)	< 0.001
**With history of cancer at baseline**				
BMI	0.970 (0.956–0.985)	< 0.001	0.975 (0.941–1.010)	0.163
WC	0.992 (0.986–0.997)	0.004	0.997 (0.984–1.011)	0.690
LBSIZ	1.196 (1.093–1.309)	< 0.001	1.378 (1.114–1.704)	0.003

Adjusted for age, sex, ethnicity/race, smoking status, alcohol consumption, history of cancer at baseline, diabetes mellitus, hypertension, dyslipidemia, and previous CVD event.

*BMI* body mass index, *WC* waist circumference, *LBSIZ* z-score of the log-transformed A Body Shape Index, *CVD* cardiovascular disease.

**Figure 2:**
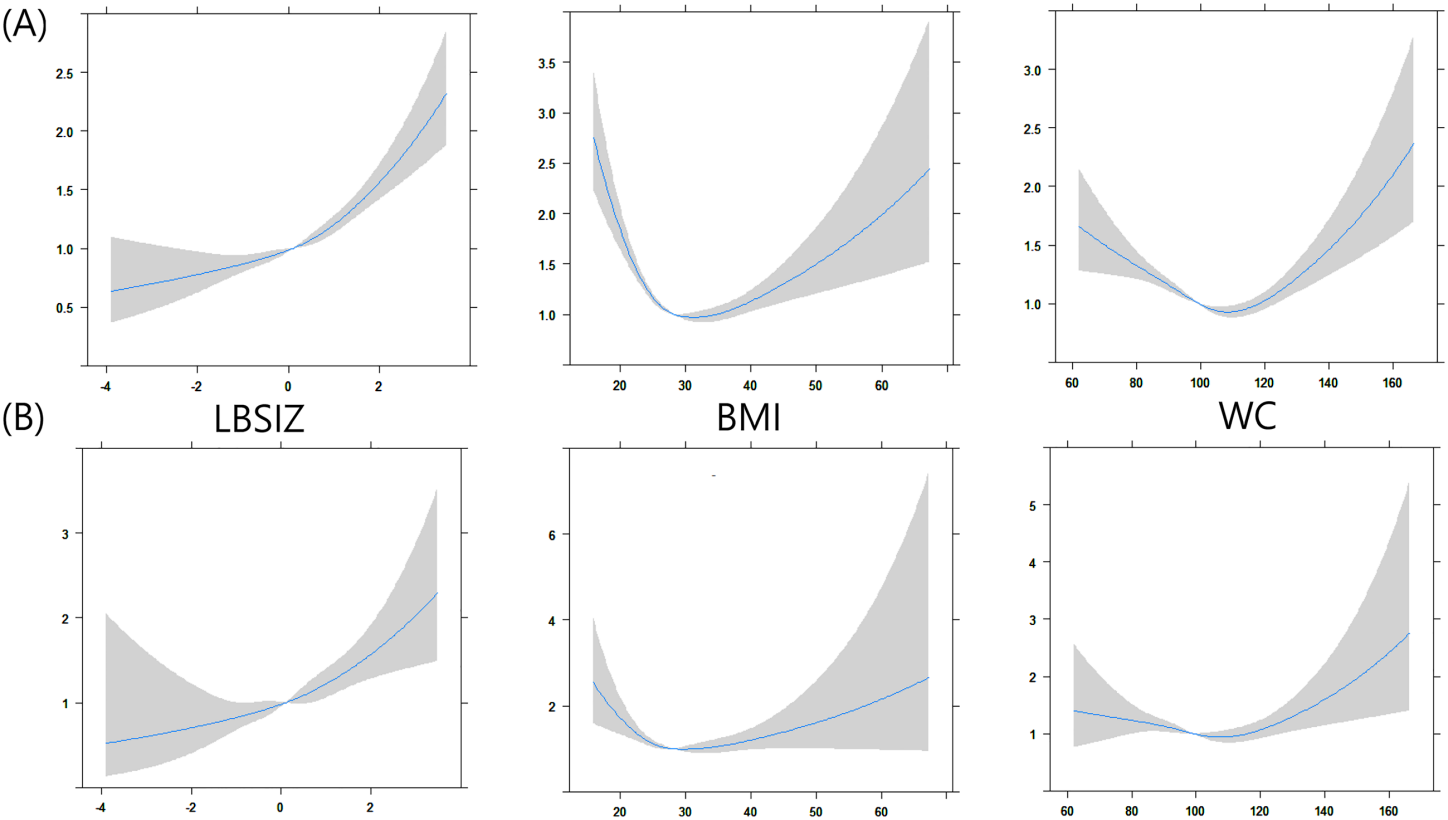
The hazard ratio for mortality according to the obesity parameters. (**A**) All-cause mortality (**B**) CVD mortality. Adjusted for age, sex, ethnicity/race, smoking status, alcohol consumption, history of cancer at baseline, diabetes mellitus, hypertension, dyslipidemia, and previous CVD event.

On the restricted cubic spline regression plot, the HR for all-cause death and CVD death increased as LBSIZ increased. This continuous increase occurred more sharply past the midpoint. However, BMI and WC showed a U-shaped association with the HR for all-cause and CVD mortalities (Fig. 2). Further analysis according to BMI classification showed that an increase in LBSIZ was positively associated with all-cause mortality in all subgroups, whereas WC was associated with all-cause mortality only for BMI ≥ 30 kg/m^2^ (Table 5).

**Table 5:** Subgroup analysis of the association between each obesity parameter and all-cause mortality according to BMI classification.

	LBSIZ		WC	
	HR (95% CI)	P-value	HR (95% CI)	P-value
Underweight (BMI < under 18.5 kg/m^2^)	1.515 (1.147–2.002)	0.003	0.996 (0.986–1.006)	0.418
Normal weight (BMI 18.5 to 24.9 kg/m^2^)	1.208 (1.121–1.302)	< 0.001	1.038 (0.981–1.098)	0.194
Overweight (BMI 25 to 29.9 kg/m^2^)	1.168 (1.091–1.249)	< 0.001	1.004 (0.996–1.013)	0.332
Obesity (BMI ≥ 30 kg/m^2^)	1.248 (1.158–1.346)	< 0.001	1.018 (1.012–1.023)	< 0.001

Adjusted for age, sex, ethnicity/race, smoking status, alcohol consumption, history of cancer at baseline, diabetes mellitus, hypertension, dyslipidemia, and previous cardiovascular disease event.

*BMI* body mass index, *WC* waist circumference, *LBSIZ* z-score of the log-transformed A Body Shape Index.

## Discussion

In this study, we investigated the association between all-cause and CVD mortalities, LBSIZ, BMI, and WC using a representative sample of the United States population. WC and BMI were positively correlated with body fat percentage and ASMI. LBSIZ was positively correlated with body fat percentage and negatively correlated with ASMI. In addition, multiple Cox regression analysis revealed that LBSIZ had significant HRs for all-cause mortality (HR: 1.22, 95% CI 1.17–1.27) and CVD mortality (HR: 1.24, 95% CI 1.14–1.36). The HR for all-cause death and CVD-related death increased as LBSIZ increased, whereas BMI and WC showed a U-shaped association.

Previous studies have reported an obesity paradox, suggesting that obesity may be associated with a decrease in mortality rate^21–23^. In a systemic review of 40 cohort studies, patients with low BMI (< 20 kg/m^2^) had a greater relative risk for total and CVD mortalities and overweight patients (BMI: 25–29.9 kg/m^2^) had a lower risk for total and cardiovascular mortalities, compared with those with normal BMI^24^. In another study, overweight (BMI 25–30 kg/m^2^) and obese (BMI 30–35 kg/m^2^) patients with hypertension and coronary artery disease had lower all-cause mortality, compared with normal weight patients (BMI 20–25 kg/m^2^)^21^. This can be explained by the fact that obesity was defined using BMI^1^, which cannot accurately distinguish fat, mineral, muscle mass, and is limited in measuring body fat^1,25–32^. Aging is associated with decreased muscle mass and increased body fat. However, BMI fails to reflect these changes and cannot assess differences in distribution of body fat by sex^26,27^. Various studies have reported an association between muscle mass and mortality^33–35^. In one cohort study conducted in Mexico, patients diagnosed with sarcopenia had a 1.39-fold higher risk of mortality^33^. In a cross-sectional study using the US NHANES data, older women with sarcopenia had an increased rate of all-cause mortality^34^. Considering the association between BMI and muscle mass^19^, many patients with low BMI are more likely to have sarcopenia, which could explain the obesity paradox^36,37^. Additionally, recent studies have reported that sarcopenic obesity is associated with an increase in mortality^38^. Elderly women with sarcopenic obesity had an increased all-cause mortality in a cross-sectional study using the US NHANES^33^. Moreover, in a meta-analysis examining prospective cohort studies, sarcopenic obesity increased the risk of all-cause mortality by 24%^39^. BMI is positively correlated with muscle mass and fat mass^19^. Thus, BMI cannot differentiate between patients with sarcopenic obesity, low muscle mass, high fat mass, and normal healthy individuals with sufficient muscle mass and low fat mass^40^. Herein, BMI was positively correlated with muscle and fat masses, and BMI and mortality risk had a U-shaped association, suggesting a paradox of obesity. As BMI has limitations, many studies have attempted to investigate obesity using other body indices, such as WC^41^. WC is closely associated with visceral fat and increased metabolic risk, morbidity, and mortality^42,43^. However, WC cannot discriminate between subcutaneous and visceral fat accumulation, and there is a lack of data related to age and sex to define obesity using WC^26,44^. In our study, similar to BMI, WC was positively correlated with muscle mass and fat mass. WC and mortality risk had a U-shaped association, suggesting a paradox of obesity.

As BMI and WC are limited, various groups of researchers have explored different indicators of obesity^41,45^. In 2012, Krakauer et al. suggested ABSI for the first time using WC, weight, and height data from 1999 to 2004 US NHANES^11^. ASBI is a more effective predictor of premature death than BMI and WC, and is an effective prognostic factor for all-cause mortality^11,46^. In a systematic review of 38 studies, including 24 retrospective cohort studies and 14 cross-sectional studies in 15 different countries, increased ABSI led to 13%, 35%, 21%, and 55% increases in the risks of hypertension, type 2 diabetes, CVD, and all-cause mortality, respectively^12^. In NHANES 1999–2004, ABSI was a strong risk factor for mortality, and similar findings were observed for mortality risk in UK samples^11,47^. Moreover, ABSI was superior to other anthropometric obesity indices, such as waist-to-height-ratio and waist-to-hip ratio^47^. Several studies have reported positive associations of ABSI with trunk fat mass^48^ and visceral adipose mass^49^, and inverse associations with fat free mass^15,19,50,51^ and hand grip strength^52^. Furthermore, A large cohort study in UK reported that body-shape phenotypes combining ABSI and Hip Index could discriminate visceral adipose tissue from abdominal subcutaneous adipose tissue^53^. Using NHANES 1999–2014, we found that an increase of 1 standard deviation in ABSI was associated with the increased risk of all-cause mortality (HR: 1.21, 95% CI 1.16–1.26), which is consistent with the result of a previous study^11^. In addition, an increase of 1 standard deviation in ABSI was associated with the increased risk of CVD-related mortality (HR 1.34, 95% CI 1.14–1.35). All the subgroup analysis according to sex and comorbidities and analysis with PSM data also showed a significant association between the z score of ABSI and all-cause or CVD-related mortality (Supplementary table 1). These results provided additional evidence of ABSI being an effective prognostic factor for all-cause or CVD-related mortality in a large population.

LBSIZ is a modified abdominal obesity scale that in its simplest form transforms logged ABSI by subtracting its mean and dividing by its standard deviation (z-score of logged ABSI) but can be readily extendable to any divided group by gender and race. LBSIZ allows an easier calculation of cut-off values and clinical application^14^. A prospective cohort study conducted in Korea reported that LBSIZ was a better predictor of CVD than BMI or WC, and that the predictability of Framingham risk score for CVD events improved using LBSIZ^18^. In another population-based study using Korean NHANES data, LBSIZ showed a linear association with CVD^54,55^. Using US NHANES, LBSIZ showed a greater association with CVD compared with other parameters of obesity^19^. Moreover, in a study of the Korean and US NHANES, LBSIZ was suggested as a cost-effective measurement tool for the prediction of low muscle mass in populations with abdominal obesity^56^. Altogether, these results suggest that LBSIZ may be a useful tool that can complement BMI.

Herein, we showed that high body shape index value is a more accurate identifying risk for CVD and mortality than BMI. This study provides evidence that LBSIZ can be used to identify high-risk groups for mortality among individuals without obesity as defined by BMI. The strength of this study is its assessment of the association between body shape and mortality using longitudinal data of a representative sample. In addition, through a detailed analysis of CVD, we observed that LBSIZ may be a significant index for primary and secondary prevention of CVD mortality. We performed a detailed analysis through PSM on age, sex, and underlying diseases based on mortality and corrected the effects of confounding variables to show an association between each obesity index and mortality.

However, some limitations must be considered. First, we could not consider the effect of weight change on mortality because of lack of data. Second, we could not evaluate the beneficial effect of improvement in LBSIZ in terms of mortality. Third, we could not conduct the analysis by racial and ethnic minority groups because we could not calculate LBSIZ for them. NHANES categorized them in the other race group, which includes Asian and multi-racial. Although the LBSIZ equation for Asians has been previously reported^19^, there is no data for the multi-racial population. Considering the heterogeneity of this group, we excluded them from this study.

## Conclusion

LBSIZ is positively correlated with all-cause and CVD mortalities. This finding is significant because it displays a different pattern from that of BMI, which showed U-shape association with mortality. Particularly, LBSIZ can be used to identify high-risk groups for mortality among individuals with low or normal BMI in both clinical practice and epidemiologic studies. Moreover, LBSIZ can compensate for the drawback of BMI, which could not distinguish muscle and fat. However, further prospective studies must be conducted to identify the beneficial effect of the improvement in LBSIZ on metabolic diseases and mortality.

## Methods

### Study population

NHANES data set from 1999 and 2014 were used in this study. Participants younger than 20 years of age with missing data (cardio-metabolic questionnaire, anthropometric or laboratory data) were excluded from the study. Out of 82,091 participants, 24,987 were included in this study (Fig. 1).

### Measurements of obesity parameters

WC was measured at the upper lateral border of the iliac crest using a measuring tape in accordance with the Centers for Disease Control and Prevention, 2012^57^. BMI was defined as the value obtained by dividing weight (kg) by square of height (kg/m^2^).

LBSIZ was calculated by standardizing the WC values according to weight and height, based on the regression of logged WC on logged weight and logged height. The residuals from the respective estimation for each race were standard normalized to make LBSIZ, which is readily extendable to any divided group by including any categorical variables for race and gender in the regression. Simple formula and details are provided in the supplementary Excel file of the previous study^14,19^.

### Measurements of covariates and outcomes

Covariates included age, sex, race/ethnicity, smoking status, alcohol consumption, and metabolic disorders, such as dyslipidemia, hypertension, diabetes mellitus, and CVD, at baseline. Data on age, sex, race/ethnicity, smoking status, and alcohol consumption were obtained using a questionnaire. Hypertension was defined as follows: systolic blood pressure > 140 mmHg, mean diastolic blood pressure > 90 mmHg, or treatment of hypertension. Diabetes using blood glucose level and HbA1c was defined as follows: fasting blood glucose level > 126 mg/dL, random blood glucose level > 200 mg/dL, HbA1c > 6.5%, or treatment of diabetes. Dyslipidemia was defined as fasting total cholesterol at 240 mg/dL or treatment of dyslipidemia.

A structured questionnaire was used to investigate the history of CVD events. Patients with one or more of the following were considered to have a history of CVD events: angina pectoris, coronary heart disease, myocardial infarction, congestive heart failure, or cerebrovascular disease^44^.

Since the DEXA data were only available from 1999 to 2006 NHANES, 11,780 participants were included in the correlation analysis between obesity parameters and body composition. Appendicular skeletal mass was defined as the sum of the total fat-free mass excluding the bone mineral content of the limbs. ASMI was calculated by dividing the appendicular skeletal mass by the square of height^2^ (m^2^). Mortality data for NHANES were obtained from public-use linked mortality files at the National Center for Health Statistics based on a probabilistic match between NHANES and the National Death Index death certificates until December 31, 2015^58^.

### Statistical analysis

Continuous and categorical variables of demographic characteristics, underlying diseases, anthropometric index, and blood test results are presented using mean with the standard deviation and frequency (%), respectively. Independent t-test and Pearson’s chi-squared test were conducted to compare the results. Pearson correlation coefficient was used to investigate the correlation between obesity parameters, ASMI, and total body fat percentage. Multiple Cox regression analysis was performed to assess the HRs for all-cause and CVD mortalities by adjusting for age, sex, race, smoking status, alcohol consumption, history of cancer, hypertension, diabetes, hyperlipidemia, and previous CVD events. Follow-up duration was calculated as the time from the first anthropometric and clinical measurements to death or last follow-up (December 31, 2015). Moreover, to control the possible bias arising from the confounding variables, PSM was carried out using a multiple logistic regression model according to the cutoff value of LBSIZ at the 75th percentile. The confounding variables selected for the PSM included age, sex, race, smoking status, alcohol consumption, history of cancer, hypertension, diabetes, hyperlipidemia, and previous CVD events. We utilized 1:2 matching by the nearest neighbor method with a caliber of 0.25 using R package “MatchIt”^20^. The graphical association between HR for each obesity parameter and mortality was evaluated using restricted cubic spline plots with four knots.

Statistical analysis was performed using International Business Machines (IBM) Statistical Package for Social Sciences version 24.0 (IBM Corporation, Armonk, NY, USA) and R version 3.1.0 (R Foundation for Statistical Computing, Vienna, Austria; www.r-project.org). P values < 0.05 were considered statistically significant.

### Ethics approval and consent to participate

The study protocol was approved by the institutional review board of Kangnam Sacred Heart Hospital (IRB No. HKS 2020-01-020). All U.S. NHANES protocols were approved by the Research Ethics Review Board of the National Center for Health Statistics, U.S. Centers for Disease Control and Prevention (NCHS IRB/ERB Protocol Number: 1999–2004, Protocol #98–12; 2005–2010, Protocol #2005–06; 2011–2016, Protocol #2011–17). All patients provided written informed consent.

## Supplementary Information

Supplementary Information.

## Data Availability

The dataset supporting the conclusions of this article is available in the CDC repository, [National Health and Nutrition Examination Survey in https://www.cdc.gov/nchs/nhanes/index.htm].
