# Supplementary material for: Association between body shape index and risk of mortality in the United States
Source: Sci Rep. 2022 Jul 4;12:11254. doi: 10.1038/s41598-022-15015-x (PMC9253149; doi:10.1038/s41598-022-15015-x)
Supplement: Supplementary file 1 — Supplementary Information. [file 41598_2022_15015_MOESM1_ESM.docx]

Supplementary Table 1. Hazard ratio (95% confidence interval) of z score of ABSI for all-cause mortality and CVD mortality.

|  | All-cause mortality | |  | CVD mortality | |
| --- | --- | --- | --- | --- | --- |
|  | HR (95% CI) | P-value |  | HR (95% CI) | P-value |
| Total | 1.211 (1.164–1.261) | <0.001 |  | 1.238 (1.136–1.349) | <0.001 |
| PSM data | 1.203 (1.154–1.254) | <0.001 |  | 1.242 (1.137–1.356) | <0.001 |
| Subgroup by sex |  |  |  |  |  |
| Men | 1.272 (1.194–1.356) | <0.001 |  | 1.211 (1.164–1.261) | <0.001 |
| Women | 1.171 (1.112–1.233) | <0.001 |  | 1.238 (1.136–1.349) | <0.001 |
| Subgroup by comorbidities at baseline |  |  |  |  |  |
| Without previous CVD events | 1.219 (1.160-1.281) | <0.001 |  | 1.313 (1.171-1.473) | <0.001 |
| With previous CVD | 1.191 (1.112-1.275) | <0.001 |  | 1.134 (0.998-1.289) | 0.054 |
| Without history of cancer at baseline | 1.218 (1.164-1.274) | <0.001 |  | 1.218 (1.107-1.340) | <0.001 |
| With history of cancer at baseline | 1.183 (1.084-1.029) | <0.001 |  | 1.351 (1.105-1.653) | 0.003 |

BMI, body mass index; WC, waist circumference; LBSIZ, z-score of the log-transformed A Body Shape Index; CVD, cardiovascular disease

Adjusted for age, sex, ethnicity/race, smoking status, alcohol consumption, history of cancer at baseline, diabetes mellitus, hypertension, dyslipidemia and previous CVD event
